# Carbon dioxide narcosis due to inappropriate oxygen delivery: a case report

**DOI:** 10.1186/s13256-017-1363-7

**Published:** 2017-07-28

**Authors:** Thomas Herren, Eva Achermann, Thomas Hegi, Adrian Reber, Max Stäubli

**Affiliations:** 1 University of Zurich, Medical Faculty, Zurich, Switzerland; 2 Department of Medicine, Limmattal Hospital, 100 Urdorferstrasse, Schlieren, ZH 8952 Switzerland; 3 Institute of Anesthesiology and Intensive Care, Limmattal Hospital, 100 Urdorferstrasse, Schlieren, ZH 8952 Switzerland; 4 University of Basel, Medical Faculty, Basel, Switzerland; 5 Department of Anesthesiology and Intensive Care, Zollikerberg Hospital, 20 Trichtenhauser Strasse, Zollikerberg, ZH 8125 Switzerland; 6 University of Bern, Medical Faculty, Bern, Switzerland; 7 Verein Komplikationenliste, Swiss Society of General Internal Medicine, 4 Lebernhöhe, Ebmatingen, ZH 8125 Switzerland

**Keywords:** Non-rebreathing mask, Oxygen inhalation therapy, Obstructive lung diseases, Hypercapnia, Respiratory acidosis

## Abstract

**Background:**

Oxygen delivery to patients with chronic obstructive pulmonary disease may be challenging because of their potential hypoxic ventilatory drive. However, some oxygen delivery systems such as non-rebreathing face masks with an oxygen reservoir bag require high oxygen flow for adequate oxygenation and to avoid carbon dioxide rebreathing.

**Case presentation:**

A 72-year-old Caucasian man with severe chronic obstructive pulmonary disease was admitted to the emergency department because of worsening dyspnea and an oxygen saturation of 81% measured by pulse oximetry. Oxygen was administered using a non-rebreathing mask with an oxygen reservoir bag attached. For fear of removing the hypoxic stimulus to respiration the oxygen flow was inappropriately limited to 4L/minute. The patient developed carbon dioxide narcosis and had to be intubated and mechanically ventilated.

**Conclusions:**

Non-rebreathing masks with oxygen reservoir bags must be fed with an oxygen flow exceeding the patient’s minute ventilation (>6–10 L/minute.). If not, the amount of oxygen delivered will be too small to effectively increase the arterial oxygen saturation. Moreover, the risk of carbon dioxide rebreathing dramatically increases if the flow of oxygen to a non-rebreathing mask is lower than the minute ventilation, especially in patients with chronic obstructive pulmonary disease and low tidal volumes. Non-rebreathing masks (with oxygen reservoir bags) must be used cautiously by experienced medical staff and with an appropriately high oxygen flow of 10–15 L/minute. Nevertheless, arterial blood gases must be analyzed regularly for early detection of a rise in partial pressure of carbon dioxide in arterial blood in patients with chronic obstructive pulmonary disease ﻿and a hypoxic ventilatory drive. These patients are more safely managed using a nasal cannula with an oxygen flow of 1–2L/minute or a simple face mask with an oxygen flow of 5L/minute.

## Background

In 2011, chronic obstructive pulmonary disease (COPD) had a global prevalence of 12% and was the third leading cause of death in the USA [1]. Oxygen (O_2_) delivery to COPD patients with an acute disease exacerbation remains challenging. High inspired O_2_ concentrations should be used with caution, because COPD patients may breathe with a hypoxic drive [2]. To avoid carbon dioxide (CO_2_) narcosis, O_2_ must be provided in a controlled fashion with a target saturation of only 88–92% [3]. This recommendation was studied in a prehospital care setting of 405 patients with COPD exacerbations: When O_2_ was delivered via nasal prongs with a target percentage of oxygen saturation of arterial blood of 88-92%, measured by pulse oximetry (SpO_2_), a significantly lower in-hospital mortality (4% versus 9%, *p* = 0.02) was observed when compared with standard O_2_ delivery using a non-rebreathing mask [4]. In addition, there were fewer hypercapnic and acidotic episodes in the group with O_2_ delivered via a nasal cannula. Accordingly, the British Thoracic Society recommends three options for oxygen delivery to COPD patients at risk for hypercapnic respiratory failure: (i) Venturi mask 28% at 4 L/min. or Venturi mask 24% at 2-4 L/min. with a target SpO_2_ of 88–92%. (ii) If the SpO_2_ falls below 88%, O_2_ delivery should be changed to a nasal cannula with an O_2_ flow 2–6 L/min. or a simple face mask with an O_2_ flow of 5 L/min. (iii) Increases in partial pressure of carbon dioxide in arterial blood (P_a_CO_2_) or progressive acidotic pH require noninvasive ventilation or intubation and mechanical ventilation [5]. Non-rebreathing masks may be used in severely hypoxic patients. It is essential to set the O_2_ flow of non-rebreathing masks to 10–15 L/min. in order to avoid CO_2_ rebreathing. Because of the risk of hypercapnic respiratory failure, blood gases must be analyzed every 30–60 minutes. We report the case of a patient with COPD who developed CO_2_ narcosis because of inadequate use of the non-rebreathing mask.

## Case presentation

**Fig. 1:**
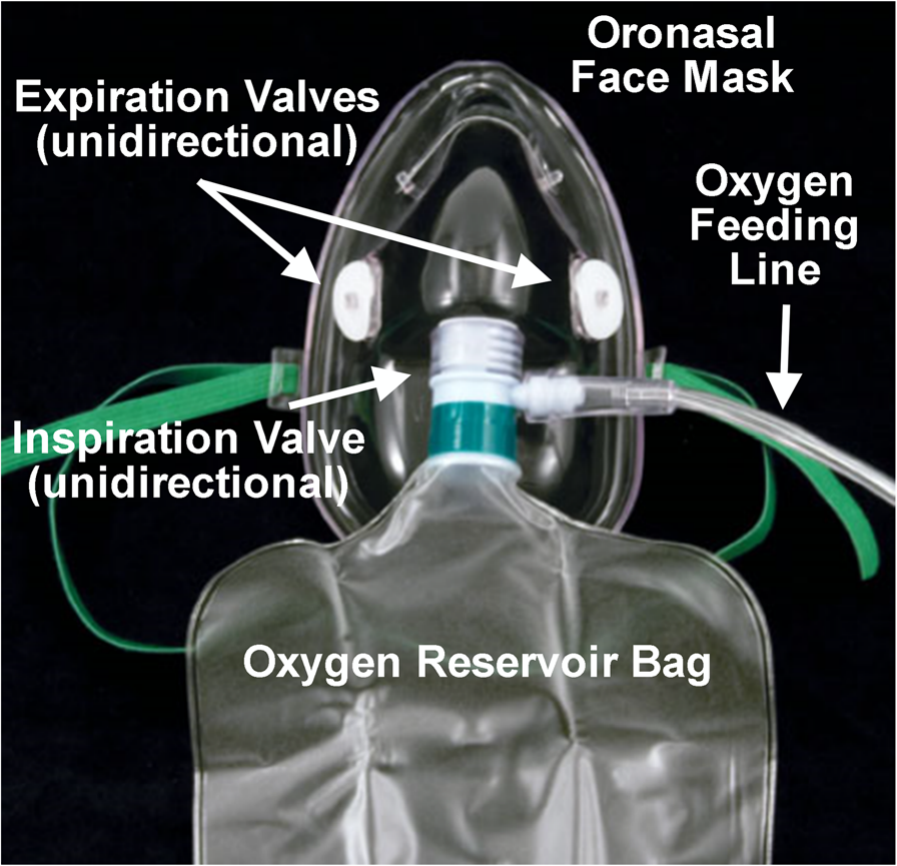
Photograph of a non-rebreathing mask with an oxygen reservoir bag attached. The mask has one unidirectional inspiration valve and two unidirectional expiration valves. The carbon dioxide exhaled by the patient is constantly diluted by a high flow of oxygen delivered to the mask (10–15 L/min.)

**Fig. 2:**
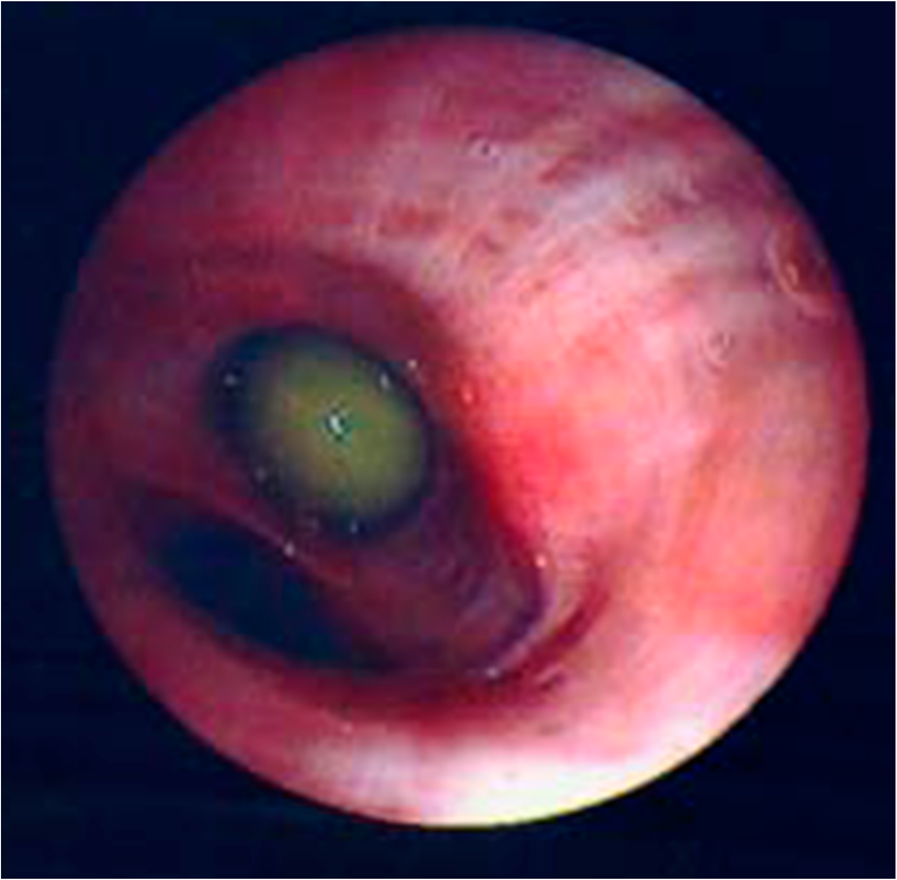
Photograph made during bronchoscopy on day 10. A pea is visible in the bronchus to the right laterobasal pulmonary segment

A 72-year-old, cachectic (height 170 cm, weight 50 kg, BMI 17.3 kg/m^2^) Caucasian man with COPD and a history of smoking had increasing shortness of breath. Pulmonary function testing performed 9 years prior showed a decreased forced expiratory volume in 1 second (FEV_1_) [38% of predicted, Global Initiative for Chronic Obstructive Lung Disease (GOLD) stage 3], which did not increase after albuterol inhalation. The high total lung capacity and residual volume were consistent with emphysema. On arrival of the emergency medical team at his home, our patient was dyspneic but alert. His vital signs were: blood pressure 150/100 mmHg, heart rate 103 bpm, and SpO_2_ 81%. High-flow O_2_ was supplied at 10 L/min. using a non-rebreathing mask with an O_2_ reservoir bag (Fig. 1). The SpO_2_ increased to 99% within 20 minutes, and our patient was transported to the emergency department. As it was unknown whether our patient had a hypoxic respiratory drive, O_2_ flow was erroneously limited to 4 L/min. He got increasingly irritated, had no headache, but was no longer oriented to time, place, and person, and became unconscious, gasping for air 2 hours later. His blood pressure was 110/80 mmHg, and the heart rate was 80 bpm. Breath sounds were distant, and his tongue was cyanotic. An arterial blood gas analysis taken shortly after starting bag-valve-mask ventilation showed marked hypercapnia with respiratory acidosis (Table 1A). Noninvasive ventilation was not a possible option [6]. Our patient was intubated, mechanically ventilated, and received albuterol and ipratropium bromide by inhalation. Methylprednisolone, amoxicillin clavulante plus clarithromycin (for an infiltrate in the right paracardiac region), and low-dose theophylline were administered intravenously. Four hours later, our patient was extubated (Table 1B), and was later transferred to a medical ward. The high P_a_CO_2_ was explained by low tidal volumes and a probably hypoxic ventilatory drive (Table 1C). Because of our patient’s worsening dyspnea, he mistakenly received O_2_ at 2 L/min. by the same non-rebreathing mask (Fig. 1). After a few hours, intensive care unit (ICU) admission was required due to hypotension (75/50 mmHg) and bradypnea (Table 1D). He was re-intubated and mechanically ventilated for 24 hours. Norepinephrine was given to stabilize the blood pressure. An electrocardiogram (ECG) showed a sinus rhythm with right bundle branch block and right ventricular hypertrophy, and the echocardiography documented a chronic cor pulmonale with pulmonary arterial hypertension. Respiratory acidosis improved, and the patient was temporarily extubated (Table 1E). However, aspiration of a pea (removed by bronchoscopy, Fig. 2) with atelectasis of the right lung again necessitated mechanical ventilation and a tracheostomy. One month after admission, the exhausted patient died of CO_2_ narcosis (Table 1F). An autopsy was not performed.

**Table 1:** Arterial blood gas analyses at different time points during the hospital stay

Time point (see text)	A, day 1	B, day 1	C, day 3	D, day 4	E, day 5	F, day 30
O_2_ flow rate; mode of administration	4 L/min.; non-rebreathing mask	2 L/min.; nasal cannula	2 L/min.; nasal cannula	2 L/min.; non-rebreathing mask	2 L/min.; nasal cannula	2 L/min.; tracheal cannula
pH (7.3–7.4)	7.1	7.3	7.4	7.2	7.4	7.1
P_a_O_2_ (kPa^a^ [11.1–14.4])	10.9	10.4	6.8	6.0	8.1	10.9
P_a_CO_2_ (kPa^a^ [4.7–6.4])	20.0	9.3	7.8	15.8	8.3	20.7
Bicarbonate (mmol/L [24–31])	41.0	37.5	35.4	42.7	36.7	49.8
Base excess (-1.5 to +3.0)	3.0	8.2	9.1	9.4	10.5	17.7
S_a_O_2_ (% [95.0–99.9])	89.4	92.5	84.8	70.3	88.6	94
Respiratory rate (L/min. [12–16])	ND	30	24	Apneic	30	ND

*ND* not determined, *O*_*2*_ oxygen, *P*_*a*_ *O*_*2*_ partial pressure of oxygen in arterial blood, *P*_*a*_ *CO*_*2*_ partial pressure of carbon dioxide in arterial blood, *p*H power of hydrogen; negative logarithm of the hydrogen ion concentration, *S*_*a*_ *O*_2_ percentage of oxygen saturation of arterial blood

^a^To convert kPa to mmHg, multiply by 7.5

## Discussion

The first two episodes of CO_2_ narcosis (Table 1A, D) were due to near asphyxia and CO_2_ rebreathing because of incorrect use of the non-rebreathing mask by providing insufficient (2–4 L/min.) O_2_ supply. The third episode was due to probable hypoventilation and severe exhaustion (Table 1F).

Assuming a reduced tidal volume [7] of 200 mL (normal 7 mL/kg body weight = 350 mL) and a respiratory rate of 30/min., our patient’s estimated minute ventilation was 6 L/minute (respiratory minute volume [mL] = tidal volume [mL] × respiratory rate [1/min.]. The respiratory minute volume [mL] is also the sum of the alveolar ventilation [mL] + the dead space ventilation [mL]). In high-concentration reservoir masks only an O_2_ flow that exceeds the minute ventilation guarantees a sufficient air supply. Consequently, the reservoir bag of the non-rebreathing mask must not be allowed to deflate by more than one third during inspiration. Our patient’s respiratory dead space was 100 mL (2 mL/kg body weight), and the dead space of the mask was 50 mL. The increased dead space (150 versus 100 mL) was relevant for gas exchange; the alveolar ventilation decreased from 3 L/min. to 1.5 L/min., which substantially compromised gas exchange. Furthermore, due to insufficient washout by the low O_2_ flow to the face mask [8], our patient rebreathed a toxic amount of CO_2_. Our patient was unable to increase his low tidal volume, and an increase in the respiratory rate was insufficient, given the considerable ventilatory dead space.

Jensen *et al.* examined the effect of different oxygen flow rates on ventilation parameters and gas exchange in ten healthy volunteers wearing a simple oronasal face mask (Hudson^®^) without a reservoir bag. Lowering the oxygen supply from 5 to 0 L/min. led to an increase in minute ventilation from 4.8 to 7.5 L/min. due to an increase in tidal volume from 380 to 540 mL with almost no change in respiratory rate. The P_a_O_2_ decreased from 38 to 13 kPa, the S_a_O_2_ from 100% to 96%, while the P_a_CO_2_ remained unchanged. The authors inferred from their results that CO_2_ retention will occur in patients with COPD wearing a simple oronasal face mask with an O_2_ supply set to < 5L/minute, because these patients cannot increase their tidal volumes [8].

In severely hypoxemic patients with COPD, O_2_ may be delivered using a non-rebreathing mask with a target O_2_ flow rate of 10–15 L/min. Arterial blood gases must be analyzed regularly. Approximately 13% of patients with COPD admitted with an exacerbation of their disease will develop CO_2_ retention during controlled O_2_ therapy [2]. In this case, it is important not to stop the O_2_ flow completely because of the risk of rebound hypoxemia [9]. For a target S_a_O_2_ of 88–92%, an O_2_ supply via nasal cannula with an O_2_ flow rate of 1–2 L/minute is usually sufficient [5]. If not, either noninvasive or mechanical ventilation must be considered [6]. Paramedics may safely use low-inspired O_2_ flow (≤4 L/min.) by nasal cannula initially in patients with COPD exacerbations [10].

## Conclusions

Non-rebreathing masks with O_2_ reservoir bags must be fed with an O_2_ flow exceeding the patient’s minute ventilation (>6–10 L/min.). If not, the amount of oxygen delivered will be too small to effectively increase the arterial O_2_ saturation. Moreover, the risk of CO_2_ rebreathing dramatically increases if the flow of oxygen to a non-rebreathing mask is lower than the minute ventilation, especially in patients with COPD and low tidal volumes. As a consequence, CO_2_ narcosis may develop. Non-rebreathing masks (with O_2_ reservoir bags) must be used cautiously by experienced medical staff and correctly with an appropriately high O_2_ flow of 10–15 L/min. [5, 11]. Nevertheless, arterial blood gases must be analyzed regularly for early detection of a rise in P_a_CO_2_ in patients with COPD and a hypoxic ventilatory drive. These patients are more safely managed using a nasal cannula with an O_2_ flow of 1–2L/min. or a simple face mask with an O_2_ flow of 5L/min. [5].

## Abbreviations

BMI: Body mass index
CO_2_: Carbon dioxide
COPD: Chronic obstructive pulmonary disease
FEV_1_: Forced expiratory volume in 1 second
GOLD: Global Initiative for Chronic Obstructive Lung Disease
O_2_: Oxygen
P_a_CO_2_: Partial pressure of carbon dioxide in arterial blood
P_a_O_2_: Partial pressure of oxygen in arterial blood
S_a_O_2_: Percentage of oxygen saturation of arterial blood
S_p_O_2_: Percentage of oxygen saturation of arterial blood, measured by pulse oximetry

## Authors’ information

MS is President of the Verein Komplikationenliste. Under the auspices of the Swiss Society of General Internal Medicine the Verein Komplikationenliste uses a program for the anonymous, systematic and prospective registration of iatrogenic complications of medical interventions in hospitalized patients in Switzerland. The patient reported was part of this registry.
